# MicroRNA -196b is related to the overall survival of patients with esophageal squamous cell carcinoma and facilitates tumor progression by regulating SOCS2 (Suppressor Of Cytokine Signaling 2)

**DOI:** 10.1080/21655979.2021.1982329

**Published:** 2021-10-04

**Authors:** Jinlong Xu, Jinmei Wang, Lili Liu, Lin Chen, Songliu Hu, Feng Liu

**Affiliations:** a Department of Cardiothoracic Surgery, Zhucheng People’s Hospital, Weifang, Shandong, China; b Department of Outpatient Operating Room, Zhucheng People’s Hospital, Weifang, Shandong, China; c Department of Radiation Oncology, Harbin Medical University Cancer Hospital, Harbin, China

**Keywords:** ESCC, miR-196b, socs2, prognosis, proliferation, migration, invasion

## Abstract

Esophageal squamous cell carcinoma (ESCC) is common cancer in China. At the same time, microRNA-196b (miR-196b) has different promotion/inhibition effects in different cancers. The study aims to reveal the role of miR-196b in ESCC and explore its prognostic value. The expression of miR-196b in ESCC samples and cell lines was detected to explore the expression pattern of miR-196b in ESCC. Kaplan-Meier method was conducted for survival rate and Multivariate Cox analysis was used to explore the clinical significance of miR-196b in ESCC. The Cell Counting Kit-8 (CCK-8) assay, transwell migration and invasion tests were used to determine the biological function of miR-196b in ESCC. The relationship of miR-196b and SOCS2 in ESCC was detected by luciferase activity assay and RIP assay. Both in ESCC tissues and cell lines, miR-196b expression was up-regulated. miR-196b expression is related to TNM stage and lymph node metastasis. Combining with the results of Multivariate Cox regression analysis, miR-196b may be a potential independent prognostic marker for ESCC patients. The results of the functional analysis showed that miR-196b inhibitor can reduce cell proliferation, migration and invasion in ESCC cells. Besides, the suppressor of cytokine signaling 2 (SOCS2) is the target of miR-196b in ESCC. miR-196b may exist as a tumor-promoting factor in ESCC and enhance the proliferation abilities, migration capacities, and invasion potential of ESCC cells by targeting SOCS2. miR-196b and SOCS2 have a close negative correlation in ESCC, which may be used as a clinically poor prognostic biomarker and therapeutic target for ESCC.

## Introduction

Esophageal squamous cell carcinoma (ESCC) occurs worldwide following the sixth leading cause of cancer mortality and ranks the 8th most common cancer [1,2]. According to the cancer statistics by the American Cancer Society, there are 18,440 new cases and 16,170 deaths in 2020 [3]. As an aggressive tumor, ESCC occurrence rates vary greatly by geographic locations, such as in north-central China and some other Asian regions following a poor five-year survival rate of less than 20% [4–6]. Currently, although tremendous progress has been made in the medical diagnosis and treatment of ESCC with the continuous development of biomedical technology, largely too late diagnosis still exists [2,5,7]. Thus, it is urgent to further identify more novel molecular biomarkers for treatment strategies.

MicroRNAs (miRNAs) are a series of non-coding short-chain (19–22 nt) RNAs that regulates gene expression at post-transcriptional levels, resulting in mRNA degradation or translation inhibition [2,8–10]. With the development of sequencing technology, miRNAs were found to influence gene expression in tumorigenesis [11–13]. Multiple aberrant expression genes were involved in the progression of ESCC, including miRNAs, such as miR-301a-3p and miR-25 [14–16]. miR-196b has unique tumor promotion/inhibition effects in many types of cancers, such as ovarian cancer, lung cancer, and laryngeal cancer [17–19]. However, the impact of miR-196b in the progression of ESCC has not been studied. There is no doubt that miR-196b sparked our great interest and we will conduct a series of investigations on the regulatory role of miR-196b in ESCC.

In this study, the clinical and functional role of miR-196b in ESCC were investigated. The expression of miR-196b and suppressor of cytokine signaling 2 (SOCS2) was measured by qRT-PCR. The luciferase activity assay found that miR-196b and SOCS2 had a negative correlation. Combined with the results of clinical statistics, we can predict that high expression of miR-196b is associated with a bad prognosis of ESCC and may become a new target for predicting the prognosis of ESCC patients.

## Materials and methods

### Patients and clinical specimens

From March 2011 to October 2015, 115 patients (mean age 58.46 ± 9.51 years) who were pathologically diagnosed by at least two histopathologists as ESCC in Harbin Medical University Cancer Hospital were included in this study. The paired ESCC tissue and adjacent normal tissue specimens were obtained during surgical resection. The tissues were immediately packaged and stored in liquid nitrogen for further use when we obtained them during the operation. The inclusion criteria were as follows: 1) the patients were pathologically diagnosed with ESCC, 2) all recruited patients had not received any additional treatment prior including radiotherapy or chemotherapy, immunotherapy, and targeted therapy. 3) patients with complete available clinical and pathological data. The complete information on lifestyle cancer risk factors and clinical data of patients were collected (Table 1 and 2). After the patients ended their hospitalization, we conducted monthly telephone five-year follow-ups to investigate the survival status of ESCC patients (Figure 2). The patient or his family members signed an informed consent form on the use of clinical samples and data on a completely voluntary basis. At the same time, our research protocol was approved by the Ethics Committee of Harbin Medical University Cancer Hospital.

**Table 1.** Relationship between miR-196b expression and clinical parameters of patients with ESCC

Variables	Cases (n = 115)	miR-196b expression		*P*-value
		Low (n = 57)	High (n = 58)	
Age				0.509
≤60	61	32	29	
>60	54	25	29	
Gender				0.768
Male	65	33	32	
Female	50	24	26	
Smoking				0.780
Absent	58	28	30	
Present	57	29	28	
Alcohol drinking				0.373
Absent	72	38	34	
Present	43	19	24	
Pickled veg consumption				0.075
Absent	49	29	20	
Present	66	28	38	
Location				0.638
Upper	23	12	11	
Middle	75	35	40	
Lower	17	10	7	
Lymph node metastasis				0.010
Negative	80	46	34	
Positive	35	11	24	
TNM stage				0.004
I–II	83	48	35	
III	32	9	23

**Table 2.** Multivariate Cox analysis of clinical characteristics in relation to overall survival

Characteristics	Multivariate analysis		
	HR	95% CI	*P*
miR-196b	2.099	1.124–3.920	0.020
Age	1.773	0.976–3.221	0.060
Gender	1.500	0.857–2.623	0.155
Smoking	1.022	0.584–1.790	0.938
Alcohold drinking	1.408	0.753–2.634	0.284
Pickled veg consumption	1.496	0.850–2.632	0.163
Location	1.592	0.624–4.059	0.330
Lymph node metastasis	1.956	1.101–3.477	0.022
TNM stage	1.846	1.060–3.215	0.030

### Cell lines and transfection

The cell lines used in this experiment including the human ESCC cell line (TE-1, Eca-109, Eca-9706, KYSE-450) and the normal esophageal epithelial cell Het-1A. TE-1, Het-1A, and Eca-9706 were obtained from the Shanghai Cell Bank of the Chinese Academy of Sciences (Shanghai, China). Eca-109 and KYSE-450 cell lines were stored in our laboratory. All cell lines are tested and certified by the company, and the cells are tested for mycoplasma every 3 months to ensure that the cells are free of contamination. These cells were incubated in Roswell Park Memorial Institute (RPMI) 1640 medium (Gibco Life Technologies, Oshima, New York, USA) containing 10% fetal bovine serum (FBS, Gibco Life Technologies). Then, the cells were placed in a 5% CO_2_ incubator at 37°C for further culture. After 24 hours, cell growth status was checked, and subculture proceeded when the cell fusion reaches 70%-80%.

Before transfection, ESCC cells were seeded in serum-free RPMI 1640 medium and incubated 24 h at 37°C when the cell was in the logarithmic growth phase. Then the cells were seeded into a 24-well plate (8x10^4^ cells/well). The miR-196b inhibitor, inhibitor NC, miR-196b mimic, and mimic NC were obtained from RIBOBIO (Guangzhou, China). Then the lipofectamine 2000 kit (Invitrogen, Carlsbad, CA, USA) was used for cell transfection experiments according to the manufacturer’s instructions. Cells were cultured for 48 h in the renewed medium and collected for follow-up experiments

### Prediction of downstream target gene

The online prediction website Targetscan (http://targetscan.org/) was used to predict the targeted binding between miR-196b and SOCS2.

### Quantitative real-time PCR assay(qRT-PCR)

RNAzol (Sigma-Aldrich) was used to isolate total RNA from ESCC tissues and cultured cell lines. 85% ethanol was used for RNA precipitation and washing. PrimeScript Reverse Transcriptase Kit (Takara) and miRNA 1st cDNA Synthesis Kit (Vazyme) were used to reverse transcription (A260/A280 ratio: 1.8 to 2.0) of RNA to cDNA. Subsequently, the expression of two types of RNA products was detected on the 7500 Real-Time PCR System (Applied Biosystems, Inc.) by using SYBR Green I Master Mix kit (Invitrogen; Thermo Fisher Scientific Inc.) and miRNA Universal SYBR qPCR Master Mix (Vazyme) respectively [20]. The 2^−ΔΔCt^ method was applied to normalize miR-196b expression to U6 level and SOCS2 expression to GAPDH level.

### Cell counting kit-8 (CCK-8) proliferation assay

The effects of miR-196b on proliferation abilities were explored in TE-1 and Eca-109 cells after transfection by using CCK-8 (Dojindo) [21]. Before the experiment, the cells were added to 96-well cell culture plates (5 × 10^3^ cells/well). Afterward, 10 μl of CCK-8 reagent (5 mg/ml; Sigma Aldrich; Merck KGaA) was added to the 96-well cell culture plate at 0, 24, 48, and 72 h time point, and then incubated at 37°C for 3 h. Finally, the absorbance of the cells was measured at 450 nm by a Varioskan LUX Multimode microplate reader (Thermo Fisher Scientific).

### Transwell migration and invasion assay

In this study, ESCC cell migration capacities and invasion power were tested by using 24-well transwell chambers (Corning Inc.) [22]. Different from the migration experiments, the invasion experiments were precoated at 37°C for 1 h with Matrigel in a liquid state. The upper chamber was added serum-free RPMI-1640 medium and the bottom chambers were added culture medium with 10% FBS. TE-1 and Eca-109 cells were added to the top chambers after adjusting the cell density to 3 × 10^4^ cells/chamber. 48 hours later, TE-1 and Eca-109 were stained with 0.1% crystal violet, and the stained cells were observed and counted through an inverted light microscope (Olympus, Japan).

### Dual-luciferase reporter assay

The sequences of SOCS2 3ʹUTR with miR-196b binding sites were synthesized and inserted into the pmirGLO luciferase reporter vector (Promega, Shanghai, China), named WT [23]. The corresponding SOCS2 mutant versions without miR-196b binding sites were synthesized and inserted into luciferase reporter vector (Promega), named MUT. Then Eca-109 cells were incubated in 12-well plates and respectively co-transfected with miR-196b mimic, mimic NC, miR-196b inhibitor, or inhibitor NC and SOCS2 3ʹUTR-WT or MUT with the help of Lipofectamine 2000 (Invitrogen). 48 hours after transfection, the firefly luciferase activities were detected by a dual-luciferase reporter assay kit (Promega).

### Western blot assay

Total protein was isolated using RIPA lysis buffer (Beyotime Institute of Biotechnology) containing protease inhibitor phenylmethanesulfonyl fluoride (Beyotime). The protein was quantitated with the BCA protein assay kit (Pierce, Rockford, IL). Then, 20 μg protein was resolved by SDS-PAGE and followed by being transferred to polyvinylidene difluoride (PVDF) membranes (Bio-Rad). The membrane was incubated with primary antibody against SOCS2 (1:1000, ab109245; Abcam) and β-actin (1:1000, ab8226; Abcam) at 4°C after blocking with 5% milk for 1 h at room temperature. Then anti-rabbit secondary antibody was used at a concentration of 1:5000 to incubate the membranes for 2 h. Finally, the protein bands were visualized using an ECL kit (Applygen, Beijing, China) and the protein levels were evaluated using ImageJ software by normalized to β-actin.

## RNA immunoprecipitation (RIP) assay

A Magna RIP RNA-Binding Protein Immunoprecipitation kit (Millipore, Billerica, MA, USA) was used in this experiment [24]. Antibodies against SOCS2 (1:50) and AGO-2 (1:50) were obtained from Abcam. The SOCS2 levels were detected using a quantitative real-time PCR assay.

### Statistical analysis

The statistical analyses were carried out using SPSS software (version 20.0; SPSS, Inc., Chicago, IL, USA) and GraphPad Prism software (version 5.0; GraphPad Software, Inc., Chicago, USA) in this study. Data are expressed as the mean ± standard deviation (SD) from triple experiments. During processing, the χ^2^ test was used to compare the association between miR-196b expression and clinic data of patients. One-way or two-way analysis of variance (ANOVA) was used to compare the significant differences among three groups and more, and Student’s t-test was used for the comparison of sample data between the two groups. Spearman’s rank correlation coefficient was used to analyze the relevance between miR-196b and SOCS2 expression levels. Kaplan-Meier analysis was used to evaluate overall survival by log-rank test and Cox regression analysis was used to determine the clinical prognosis significance of miR-196b. The difference was considered statistically significant for *P*-value less than 0.05.

## Results

This study aimed to investigate the role of miR-196b in ESCC. Firstly, the expression of miR-196b was measured in ESCC tissues and cell lines using a quantitative real-time PCR assay. The results revealed that miR-196b was upregulated in ESCC and associated with clinical characteristics, suggesting that miR-196b expression may play an oncogenic role and be involved in the progression of ESCC. Moreover, clinical analysis results indicated that miR-196b was associated with shorter overall survival outcomes. Additionally, miR-196b may accelerate tumor cell proliferation, migration, and invasion by targeting SOCS2.

### Expression of miR-196b in ESCC

**Figure 1.**
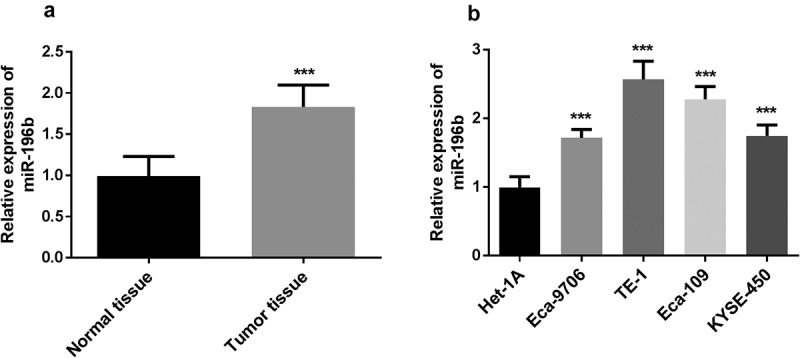
miR-196b expression was measured in ESCC tissue and cells. A. miR-196b expression was increased in ESCC tissue samples compared to adjacent normal tissue specimens. the analysis was carried out using paired students’ t-test by graphpad prism software. B. miR-196b expression was raised in four ESCC cell lines than normal esophageal epithelial cell Het-1A. the data were analyzed using one-way ANOVA by graphpad prism software. ***P < 0.001

In the experiment, the expression levels of miR-196b were detected using qRT-PCR. First, the expression level of miR-196b was measured in 115 pairs of ESCC tissues and adjacent normal tissues. The results manifested that the expression levels of miR-196b in tumor tissues were increased (Figure 1(a), *p* < 0.001). Then, as shown in (Figure 1(b)), the expression levels of miR-196b in all ESCC cell lines were significantly up-regulated (all *P* < 0.001). The above results adequately indicate that miR-196b is overexpressed in ESCC. Besides, the expression levels of miR-196b were increased significantly in the TE-1 cell line, followed by the Eca-109 cell line (*P* < 0.001). Since both TE-1 and Eca-109 cell lines have similar higher miR-196b expression levels, both are used in subsequent experiments.

**Figure 2.**
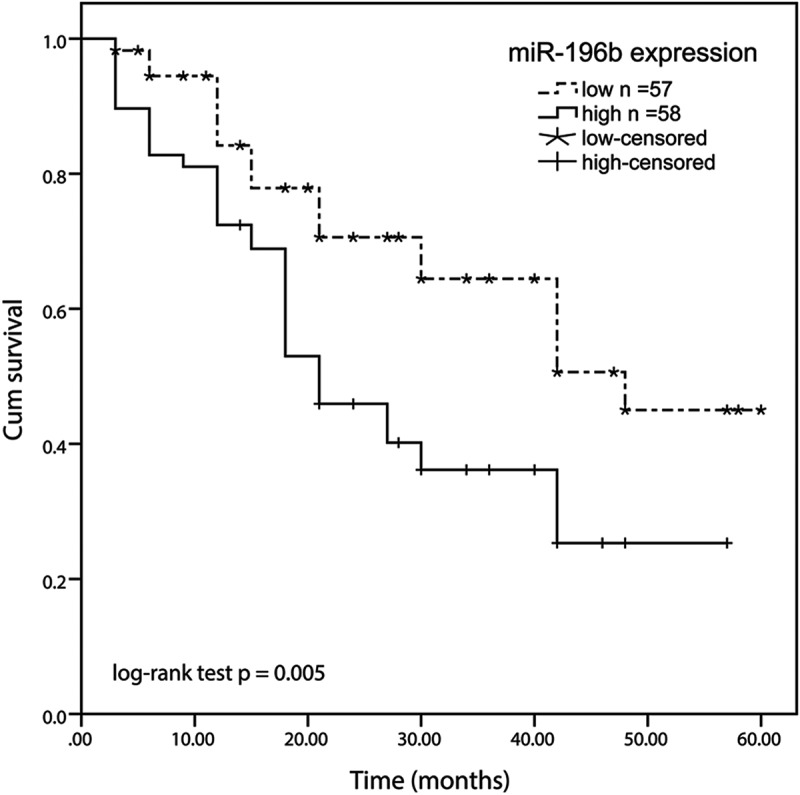
Kaplan-meier method was conducted to analyze the five-year survival rate of ESCC patients using SPSS software. high expression of the miR-196b group displayed a shorter overall survival rate than the low miR-196b expression group (log-rank test *P*= 0.005)

### Increased expression of miR-196b was relevant to clinical features of patients with ESCC

Besides, the relationship between the expression level of miR-196b and the clinical characteristics of ESCC patients was shown in Table 1. In line with the average expression value of miR-196b as the critical value, all patients were divided into low expression groups (57 patients) and high expression groups (58 patients). Through the χ^2^ test, the expression of miR-196b has no connection with gender, age, smoking, alcohol drinking, pickled vegetable consumption, and location with esophageal squamous cell carcinoma (all *P* > 0.05), but it is closely related to the TNM stage (*P* = 0.010) and lymph node metastasis (*P* = 0.004)

### miR-196b has a prognostic value in ESCC

Kaplan-Meier analysis was used to evaluate the overall survival rate of patients with ESCC in both miR-196b expression groups in this experiment. We made a 5-year overall survival rate according to the correlation between the expression level of miR-196b and the overall survival information of ESCC. The results exhibited that the survival rate of ESCC patients in the low miR-196b expression group was higher than the high miR-196b expression group (log-rank test *P* = 0.005, Figure 2). Besides, multivariate Cox’s clinical characteristics analysis results showed that the miR-196b expression (HR = 2.099, 95% CI = 1.124–3.920, *P* = 0.020) was an independent prognostic risk factor (Table 2). Based on the above results, miR-196b may be an independent poor prognostic marker for ESCC.

### miR-196b weakened ESCC cell proliferation, migration, and invasion

**Figure 3.**
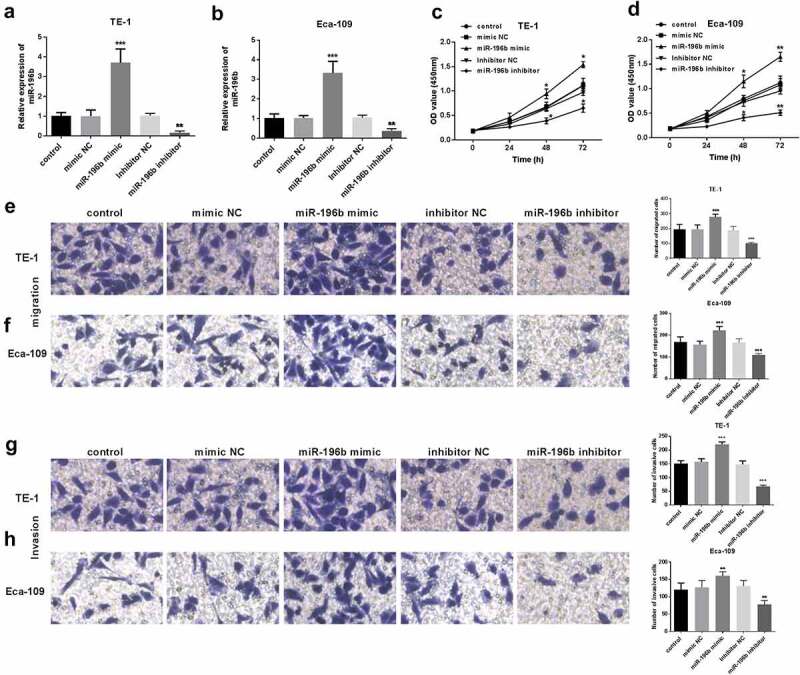
Proliferation of TE-1 and Eca-109 cells was facilitated or suppressed after the upregulation or downregulation of miR-196b compared to untreated cells. (a) and (b) expression level of miR-196b was determined in TE-1 and Eca-109 cells by qRT-PCR after transfection with miR-196b mimic, inhibitor, or NCs. (c) and (d) proliferative capacity of TE-1 and Eca-109 cells were measured by CCK-8. (e) and (f) Migratory ability of TE-1 and Eca-109 cells were measured by transwell assay (magnification 200×). (g) and (h) Invasive ability of TE-1 and Eca-109 cells were measured by transwell assay (magnification 200×). The above analysis was performed using one-way ANOVA by graphpad prism software. *P < 0.05, ***P < 0.001

The biological functions of miR-196b in ESCC were further evaluated. First, qRT-PCR was performed to explore whether miR-196b expression was upregulated or inhibited when miR-196b mimics or inhibitors were transfected into TE-1 and Eca-109 cells. As shown in (Figure 3(a-b)) the expression of miR-196b was significantly reduced in TE-1 and Eca-109 cells transfected with miR-196b inhibitor, while was elevated after transfection of miR-196b mimic (all *P*< 0.05). Then, CCK-8 analysis was implemented to determine the proliferation ability of the TE-1 and Eca-109 cells. The results showed that the cell that was transfected with miR-196b mimic showed enhanced proliferation while miR-196b inhibitor reduced proliferation ability (*P* < 0.001, Figure 3(c-d)). Also, the results of the transwell migration and invasion test showed that miR-196b inhibitors caused the decreased migration and invasion ability of TE-1 and Eca-109 cells but miR-196b mimics caused the enhanced migration and invasion (all *P*< 0.001, Figure 3(e-h)). According to the above experiments, miR-196b inhibitor can suppress the proliferation potential, migration, and invasion abilities of cells, in other words, increased expression of miR-196b can promote ESCC cells’ cellular activities.

### SOCS2 is a target of miR-196b in ESCC

**Figure 4.**
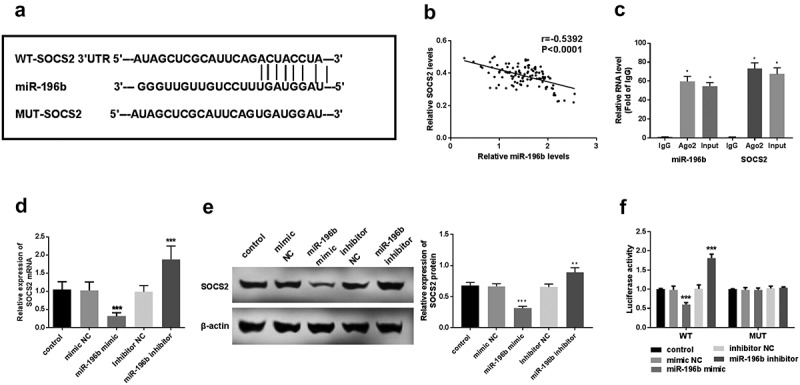
miR-196b interacted with *SOCS2*. (a) the binding site between SOCS2 and miR-196b is shown. (b) spearman correlation analysis revealed the negative correlation between miR-196b and *SOCS2* expression. (c) the cell lysate was incubated with an anti-AGO2 antibody for RIP, and the SOCS2 content was measured by RT-PCR. (d) expression of *SOCS2* mRNA was detected using qRT-PCR in TE-1 cells with upregulated or downregulated expression of miR-196b. (e) the expression of SOCS2 protein was measured using a western blot assay. (f) luciferase activity of WT-*SOCS2* TE-1 cells group was weakened or enhanced by miR-196b overexpression or knockdown, respectively, but luciferase activity has no change in MUT-*SOCS2* TE-1 cells by miR-196b. The above analysis was performed by graphpad prism software.***P < 0.001

First, using an online prediction website to explore whether miR-196b and SOCS2 can interact in ESCC. The prediction results showed that SOCS2 and miR-196b can form multiple bases pairing in and the binding sites between SOCS2 3ʹUTR and miR-196b were displayed in (Figure 4(a)). Spearman’s rank correlation coefficient analyzed the relevance between miR-196b and SOCS2 expression levels. The results revealed that miR-196b and SOCS2 expression have a significant negative correlation (r = −0.5392, *P* < 0.0001, Figure 4(b)). qRT-PCR was used to determine the gene expression level of SOCS2 in TE-1 cells. SOCS2 expression was elevated in anti-AGO2-incubated TE-1 cells (*P* < 0.05, Figure 4(c)). It was found that in the case of miR-196b inhibitor transfection, the expression level of SOCS2 was significantly up-regulated, while in the cases of miR-196b mimic transfection, SOCS2 mRNA and protein expression was downregulated (*P* < 0.01, Figures 4(d-e)). Finally, we used TE-1 cells with a particularly high or low expression of miR-196b to detect luciferase activity. The luciferase assay results indicated the miR-196b mimics or inhibitors does not affect the luciferase activity of MUT transfected cells, while in WT transfected cells, elevated expression of miR-196b reduces the luciferase activity, on the contrary, knockdown of miR-196b increased the luciferase activity of TE-1 cells (*P* < 0.001, figure 4(f)). This indicates that miR-196b may bind to the predicted site and then inhibit the expression of SOCS2.

## Discussion

ESCC is a common malignant tumor of the upper gastrointestinal tract, and China is a high prevalence area for ESCC [6,25]. Despite advances in esophageal cancer surgery and radiotherapy, the overall survival rate of patients has not improved and the 5-year survival rate remains at a low level [26]. The reason for this may be a combination of the lack of timely ESCC detection and limited advanced treatment. Therefore, it is imperative to seek new predictors and therapeutic target sites for ESCC. Our objective was to investigate the previously unfound new role whereby miR-196b acts in ESCC.

According to the data obtained from the qRT-PCR experiment, miR-196b expression in the tumor tissue specimens of ESCC patients was increased. The expression of miR-196b in the four esophageal squamous cell carcinoma cell lines was also high, which was in keeping with previous studies where miR-196b was up-regulated in the non-small cell lung cancer, ovarian cancer, gastric cancer, breast cancer, and colon cancer [17,27–30]. However, there are differences in some cancers about the miR-196b expression. miR-196b is low expressed in liver cancer, cervical cancer, and melanoma, suggesting that it may also be a tumor suppressor [31–34]. Thus, miR-196b may play different roles in cancers based on different cancer types by targeting downstream genes. According to this study, miR-196b may be overexpressed and promote the progression of ESCC.

Next, the role of miR-196b in ESCC was probed and evaluated based on clinical experimental statistics. The abnormal expressions of miR-196b were associated with different overall survival results. Among them, the prognosis living situation of the high miR-196b expression group was worse than the low miR-196b expression group. In clinical analysis, miR-196b expression is related to the positive lymph node metastasis and high TNM stage of ESCC. Besides, miR-196b expression is an independent prognostic indicator of ESCC. In summary, miR-196b may have a prognostic value for ESCC. The prognostic value of high miR-196b expression was also observed in ovarian cancer [35], which confirmed the prognostic value of miR-196b in cancers.

Functional experiments were carried out to assess the influence on cell function of miR-196b by transfected miR-196b mimic/inhibitor in TE-1 and Eca-109 cells. The results uncovered that increased expression of miR-196b can accelerate tumor cell proliferative abilities, migratory potential, and invasive capacities, while miR-196b inhibitor can significantly inhibit the proliferation, migration, and invasion of ESCC cells. A similar finding was observed in the current study that transfection with miR-196b inhibitor can reduce the proliferation of ovarian cancer cells [17]. Therefore, we speculate that miR-196b may accelerate the proliferation, migration, and invasion of ESCC cells and play a promotion role in ESCC progression.

So far, a series of miR-196b direct regulatory genes have been verified, such as SOCS2 [19], homeobox C8 (HOXC8) [29], transcription factor GATA-6 (GATA6) [18], Homeobox Protein HOX-B7 (HOX-B7) and BMP4 [33]. For instance, increased expression of miR-196b inhibits SOCS2 in laryngeal squamous cell carcinoma resulting in tumor progression and poor prognosis outcomes [19]. In these studies, miR-196b played a variety of roles in different tumor cells by regulating different target genes. SOCS2 plays a tumor-suppressor role in several cancers [36–38]. In this study, we determined the relationship between SOCS2 and miR-196b in ESCC. In the experiment, we first used the online prediction website and predicted that SOCS2 gene sequences have binding sites with miR-196b. At the same time, Spearman’s rank correlation coefficient analysis showed that miR-196b is closely related to *SOCS2*, and miR-196b levels negatively correlated with *SOCS2* expression. RIP results confirmed the direct interaction between miR-196b and SOCS2. Then, the SOCS2 mRNA and protein expression levels were downregulated or up-regulated when the ESCC cell was transfected with miR-196b mimic or inhibitor, respectively. The dual-luciferase reporter assay revealed that the luciferase activity of MUT transfected cells was not affected by the expression of miR-196b inhibitor or mimic, in turn, the luciferase activity of the transfected cells was significantly reduced by the miR-196b mimic in WT transfected cells, while was significantly increased by miR-196b inhibitor. Our research revealed that miR-196b may also drive ESCC cell proliferation, migration, and invasion by targeting *SOCS2*.

## Conclusion

In summary, the high expression of miR-196b in ESCC can promote the process of ESCC. On this basis, miR-196b can be used as an independent poor prognostic molecule of ESCC, and ESCC patients with high expression of miR-196b have a poor prognosis. At the same time, miR-196b may promote ESCC proliferation by targeting *SOCS2*. This experiment may give miR-196b the possibility of clinical application as a potential target.

## References

[1] Sakai NS, Samia-Aly E, Barbera M, et al. A review of the current understanding and clinical utility of miRNAs in esophageal cancer. Semin Cancer Biol. 2013 23(6):512–521. 24013023 10.1016/j.semcancer.2013.08.005

[2] Yang H, Su H, Hu N, et al. Integrated analysis of genome-wide miRNAs and targeted gene expression in esophageal squamous cell carcinoma (ESCC) and relation to prognosis. BMC Cancer. 2020;20(1):1. 10.1186/s12885-020-06901-6 PMC7201714 32375686

[3] Siegel RL, Miller KD, Jemal A. Cancer statistics, 2020. CA Cancer J Clin. 2020;70(1):7-30. 10.3322/caac.21590 31912902

[4] Fakhrian K, Ordu AD, Lordick F, et al. Long-term outcomes of trimodality treatment for squamous cell carcinoma of the esophagus with cisplatin and/or 5-FU. Strahlentherapie Und Onkologie. 2014;190(12):1133–1140. 25015426 10.1007/s00066-014-0711-4

[5] Tang WR, Chen ZJ, Lin K, et al. Development of esophageal cancer in Chaoshan region, China: association with environmental, genetic and cultural factors. Int J Hyg Environ Health. 2015;218(1):12–18. 25455641 10.1016/j.ijheh.2014.10.004

[6] Chen W, Zheng R, Baade PD, et al. Cancer statistics in China, 2015. CA Cancer J Clin. 2016;66(2):115–32. 26808342 10.3322/caac.21338

[7] Chen Q, Hou J, Wu Z, et al. miR-145 Regulates the sensitivity of esophageal squamous cell carcinoma cells to 5-FU via targeting REV3L. Pathol Res Pract. 2019;215(7):7. 10.1016/j.prp.2019.04.019 31072625

[8] Roush SF, Slack FJ. Micromanagement: a role for microRNAs in mRNA stability. ACS Chem Biol. 2006;1(3):132–134. 17163657 10.1021/cb600138j

[9] dev.m, Bartel DP. MicroRNAs: target recognition and regulatory functions. Cell. 2009;136(2):215–33 10.1016/j.cell.2009.01.002 PMC3794896 19167326

[10] Ohashi S, Miyamoto S, Kikuchi O, et al. Recent Advances From Basic and Clinical Studies of Esophageal Squamous Cell Carcinoma. Gastroenterology. 2015;149(7):1700–15 26376349 10.1053/j.gastro.2015.08.054

[11] Ambs S, Prueitt RL, Yi M, et al. Genomic profiling of microRNA and mRNA reveals deregulated microRNA expression in prostate cancer. other. 2008;68:15. 10.1158/0008-5472.CAN-08-0144 PMC2597340 18676839

[12] Iorio MV, Croce CM. MicroRNAs in cancer: small molecules with a huge impact. J Clin Oncol. 2009;27(34):5848–56. 19884536 10.1200/JCO.2009.24.0317 PMC2793003

[13] Flynt AS, Lai EC. Biological principles of microRNA-mediated regulation: shared themes amid diversity. Natrevgenet. 2008;9(11):831. 10.1038/nrg2455 PMC2729318 18852696

[14] Zhao Y, Huang J, Chen J. The integration of differentially expressed genes based on multiple microarray datasets for prediction of the prognosis in oral squamous cell carcinoma. cell. 2021 Dec;12(1):3309–3321. 10.1080/21655979.2021.1947076 PMC8806768 34224327

[15] Zhang N, Liu JF. MicroRNA (MiR)-301a-3p regulates the proliferation of esophageal squamous cells via targeting PTEN. Bioengineered. 2020 Dec;11(1):972–983. 32970954 10.1080/21655979.2020.1814658 PMC8291791

[16] Liu B, Li X, Li C, et al. miR-25 mediates metastasis and epithelial-mesenchymal-transition in human esophageal squamous cell carcinoma via regulation of E-cadherin signaling. Bioengineered. 2019;10(1):679–688. 31679450 10.1080/21655979.2019.1687391 PMC8530270

[17] Li Y, Li J, Liu Z, et al. High Expression of miR-196b Predicts Poor Prognosis in Patients with Ovarian Cancer. Onco Targets Ther. 2020;13:9797–9806. 33061458 10.2147/OTT.S254942 PMC7534859

[18] Li H, Feng C, Shi S. miR-196b promotes lung cancer cell migration and invasion through the targeting of GATA6. Oncol Lett. 2018;16(1):247–252. 10.3892/ol.2018.8671 PMC6006457 29928408

[19] Zhao X, Zhang W, Ji W. miR-196b is a prognostic factor of human laryngeal squamous cell carcinoma and promotes tumor progression by targeting SOCS2. Biochem Biophys Res Commun. 2018 Jun 22;501(2):584–592. 29753737 10.1016/j.bbrc.2018.05.052

[20] Yu H, Ding X, Shang L, et al. Protective Ability of Biogenic Antimicrobial Peptide Microcin J25 Against Enterotoxigenic Escherichia Coli-Induced Intestinal Epithelial Dysfunction and Inflammatory Responses IPEC-J2 Cells. Front Cell Infect Microbiol. 2018;8:242. 30057893 10.3389/fcimb.2018.00242 PMC6053529

[21] Jing H, Qu X, Liu L, et al. A Novel Long Noncoding RNA (lncRNA), LL22NC03-N64E9.1, Promotes the Proliferation of Lung Cancer Cells and is a Potential Prognostic Molecular Biomarker for Lung Cancer. Med Sci Monit. 2018 Jun 23;24:4317–4323. 29935018 10.12659/MSM.908359 PMC6047588

[22] Han B, Ge Y, Cui J, Liu B. Down-regulation of lncRNA DNAJC3-AS1 inhibits colon cancer via regulating miR-214-3p/LIVIN axis. Bioengineered. 2020;11(1):524–535. 32352854 10.1080/21655979.2020.1757224 PMC7202691

[23] Zhang H, Peng J, Lai J, et al. MiR-940 promotes malignant progression of breast cancer by regulating FOXO3. Biosci Rep. 2020 Sep 30;40(9):9. 10.1042/BSR20201337 PMC7494982 32840296

[24] Zhao Z, Liang S, Sun F. LncRNA DLX6-AS1 Promotes Malignant Phenotype and Lymph Node Metastasis in Prostate Cancer by Inducing LARGE Methylation. Front Oncol. 2020;10:1172. 32850336 10.3389/fonc.2020.01172 PMC7424052

[25] Lin Y, Totsuka Y, He Y, et al. Epidemiology of Esophageal Cancer in Japan and China. J Epidemiol. 2013;23(4):233. 23629646 10.2188/jea.JE20120162 PMC3709543

[26] Torre LA, Bray F, Siegel RL, et al. Global cancer statistics, 2012. Ca A Cancer J Clinicians. 2015;65(2):87–108. 10.3322/caac.21262 25651787

[27] V?sa U, Vooder TN, Kolde R, et al. Identification of MiR-374a as a prognostic marker for survival in patients with early-stage nonsmall cell lung cancer. Genes Chromosomes & Cancer. 2011;50(10):812–822. 21748820 10.1002/gcc.20902

[28] Woo LS, Cheol PK, Goo KJ, et al. Dysregulation of MicroRNA-196b-5p and MicroRNA-375 in Gastric Cancer. J Gastric Cancer. 2016;16(4):221–229. 28053808 10.5230/jgc.2016.16.4.221 PMC5206312

[29] Li Y, Zhang M, Chen H, et al. Ratio of miR-196s to HOXC8 mRNA Correlates with Breast Cancer Cell Migration and Metastasis. Cancer Res. 2010;70(20):7894–7904. 20736365 10.1158/0008-5472.CAN-10-1675 PMC2955846

[30] Ge J, Chen Z, Li R, et al. Upregulation of microRNA-196a and microRNA-196b cooperatively correlate with aggressive progression and unfavorable prognosis in patients with colorectal cancer. Cancer Cell Int. 2014;14(1):1–8. 25525411 10.1186/s12935-014-0128-2 PMC4269845

[31] Rebucci M, Sermeus A, Leonard E, et al. miRNA-196b inhibits cell proliferation and induces apoptosis in HepG2 cells by targeting IGF2BP1. Mol Cancer. 2015 APR 08;14(1):79. 25889892 10.1186/s12943-015-0349-6 PMC4403945

[32] Micevic G, Muthusamy V, Damsky W, et al. DNMT3b Modulates Melanoma Growth by Controlling Levels of mTORC2 Component RICTOR. Cell Rep. 2016;14(9):9. 10.1016/j.celrep.2016.02.010 PMC4785087 26923591

[33] Braig S, Mueller DW, Rothhammer T, et al. MicroRNA miR-196a is a central regulator of HOX-B7 and BMP4 expression in malignant melanoma. Cell Mol Life Sci. 2010;67(20):3535–3548. 20480203 10.1007/s00018-010-0394-7 PMC11115699

[34] Christine H, Hui ABY, Alajez NM, et al. MicroRNA-196b Regulates the Homeobox B7-Vascular Endothelial Growth Factor Axis in Cervical Cancer. Plos One. 2013;8(7):e67846. 23861821 10.1371/journal.pone.0067846 PMC3701631

[35] Li Y, Li J, Liu Z, et al. High Expression of miR-196b Predicts Poor Prognosis in Patients with Ovarian Cancer. Onco Targets Ther. 2020;8:9797–9806. 10.2147/OTT.S254942 PMC7534859 33061458

[36] Letellier E, Schmitz M, Baig K, et al. Identification of SOCS2 and SOCS6 as biomarkers in human colorectal cancer. Br J Cancer. 2014 Aug 12;111(4):726–735. 25025962 10.1038/bjc.2014.377 PMC4134506

[37] Shi LP, Liang M, Li FF, et al. MiR-492 exerts tumor-promoting function in prostate cancer through repressing SOCS2 expression. Eur Rev Med Pharmacol Sci. 2019 Feb;23(3):992–1001. 30779065 10.26355/eurrev_201902_16986

[38] Xu J, Chen Q, Tian K, et al. m6A methyltransferase METTL3 maintains colon cancer tumorigenicity by suppressing SOCS2 to promote cell proliferation. Oncol Rep. 2020 Sep;44(3):973–986. 32705223 10.3892/or.2020.7665 PMC7388248

